# Self-referral patterns among federal civil servants in oyo state, South-Western Nigeria

**DOI:** 10.11604/pamj.2017.26.105.11483

**Published:** 2017-02-28

**Authors:** Henry Okoli, Taiwo Obembe, Kayode Osungbade, Folashayo Adeniji, David Adewole

**Affiliations:** 1 Department of Health Policy and Management, Faculty of Public Health, College of Medicine, University of Ibadan, Ibadan, Nigeria

**Keywords:** Self-referral, federal civil servants, primary health care, knowledge

## Abstract

**Introduction:**

Primary health care is widely accepted as the first point of care; yet, individuals requiring healthcare engage in self-referrals to higher levels of care thereby by-passing primary care. Little is known of the extent to which self-referrals are carried out when care is needed. This study thus sought to determine the prevalence of self-referral, its patterns and factors influencing self-referrals amongst federal civil servants in Southwestern Nigeria.

**Methods:**

A cross-sectional study was carried out among 300 federal civil servants who were interviewed using validated and pre-tested interviewer-administered semi structured questionnaires. Data was analyzed using univariate and Chi-square test at level of significance set at P <0.05.

**Results:**

Mean age of the respondents was 39.96 ± 9.1 years with majority being married (80.7%); 90.7% completed tertiary education (and 76.7 % were middle grade (7-12) level officers. Most (60.0%) of the respondents had ever engaged in self-referral. Malaria was the commonest health problem (39.7%) for self-referral to secondary or tertiary facilities. Desire for quality service (35.7%) and competent staff (35.2%) were the commonest reasons for self-referral to a higher level of health care. More female respondents (76.0%) compared to male respondents (64.0%) significantly engaged in self-referral (p = 0.02, X2 = 5.14). Respondents having good knowledge of referral practices engaged less in self-referral compared to those with poor knowledge. (p = 0.02, X2 = 5.43).

**Conclusion:**

Having good knowledge of referral practices and being male are positively associated with referral practices. Creating awareness and improving knowledge on referral practices with special emphasis on women population are desirable strategies for encouraging the use of primary health care as first of point of contact with health systems.

## Introduction

Prior to independence, millions of people on the geographic or social periphery of African countries received either marginal health care or none at all [1]. Colonial health systems channelled resources to urban dwellers and white settlers at the expense of the predominantly black rural populations who had the greatest need [1]. Many years after independence, Nigeria instituted Primary Health Care (PHC) systems, and developed a pyramidal referral model to support the primary health care level. Clinics and health centres were intended to provide local services for uncomplicated cases, referring patients with more serious conditions to state and teaching hospitals [2]. The Primary Health Care is designed to provide general health services for preventive, curative, promotive and rehabilitative purposes to the population; serving as the entry point of the health care system, while the secondary and tertiary health care renders specialized and highly specialized services respectively to patients that are referred from the primary health care facilities [3]. The development of effective patient referral systems is germane to effective management, prognosis and outcomes of ailments in the hospital setting. “Referral is a process by which a health worker transfers the responsibility of care temporarily or permanently to another health professional or social worker or to the community” [4]. Referral has also been defined as a process in which a health worker at one level of the health system, having insufficient resources (drugs, equipment, skills) to manage a clinical condition, seeks the assistance of a better or differently resourced facility at the same or higher level to assist in, or take over the management of, the client's case [5]. Referral systems have been considered to be an important component of health systems in developing countries since the emergence of primary healthcare [6]. In practice, referrals are not only between lower and higher-level facilities (considered to be an integral part of allopathic health systems) [6], but also between primary facilities as well as within hospitals which can be vertical, horizontal or diagonal [7]. The referral system thus ensures a continuum of care as patients are required to seek health services from higher tiers of care in the referral system should there be need to do so [8]. Furthermore, it ensures that patients are dealt with in the right place with effective treatment provided at the affordable cost [9]. Advantages of an effective referral system in literature have been also documented to contribute to high standards of care by limiting over-medicalization, permitting an efficient division of tasks between generalists and specialists, freeing specialists to develop their special skills, and by reducing medical cost [10]. Literature has extensively documented the referral of patients within hospital levels both in Nigeria [4] and abroad [11]. Irrespective of the reasons that range from cheaper hospital out-patient services to improved quality [11], self-referral has generated economic concerns and is a practice that should be discouraged if its cons outweighs its pros [12, 13]. A recent study conducted in Ilorin found out that only 7.1% were referred from primary health centres to the hospital while 91.9% reported directly without referral [14]. The importance of referral systems has been documented in several studies; however there is a dearth of literature on self-referrals particularly in sub-Saharan Africa notable for self-referrals. This study sought to add to existing body of knowledge by examining patterns of self-referral practices among civil servants. This especially becomes more important in a setting such as Nigeria, a low and middle income country (LMIC), where the health system is not as optimal as that of developed countries. Findings from this study will help to inform health policy decisions geared at strengthening of the health system which comes with improvement on the referral linkage.

## Methods

This descriptive cross-sectional study was conducted between October and November, 2014 at the Federal Secretariat Complex, Ikolaba, and Ibadan, Nigeria. Ibadan is the ancient capital city of Oyo State, located in the south-west of Nigeria. Ibadan city is divided into eleven local government areas, 5 in the metropolis and 6 in the peri-urban area [15]. Ibadan is a centre of trade and farming, renowned for its production of cocoa, palm oil, yams, cassava, corn and fruits. The inhabitants are mainly Yorubas, with clusters of Igbos and Hausas living in several areas [16]. The population under study were the Federal civil servants working at the Federal Secretariat Complex, Ikolaba, and Ibadan. There are 8 ministries and 13 agencies located within the secretariat with staff strength of 853 working within the complex as at April 2014. A minimum sample size of 282 was calculated using 5% level of significance and 24.3% as the proportion of those that by-passed health centre to seek treatment at tertiary facility [17]. The study included all Federal civil servants working within the secretariat who have been employed for at least one year as at the time of the study but excluded all temporary staff or contract staff under the employment of any of the agencies or ministries. A total of 354 federal workers met the inclusion criteria across all the 8 ministries and 13 agencies and thus were invited to participate in the study; out of these, 300 respondents consented to participate (Response Rate = 84.7%). Two repeat visits were conducted to enrol those who were absent from the secretariat during the initial data collection. Data was collected with the aid of a pre-tested and validated interviewer-administered semi-structured questionnaire. The questionnaire was pre-tested among 30 civil servants in the Local Government Civil Service Commission at Ibadan North-East. The pre-tested questionnaire was analysed and necessary modifications effected. Three research assistants with completed secondary school education were trained on data collection. Knowledge of the referral system was scored based on response to 5 questions. Zero was scored for “not supported”, 1 for “indifference”, and 2 for “support”. A score range of 0-5 was considered poor knowledge while 6-10 was considered good knowledge. Perception of the effectiveness of the referral system was measured and scored using 21 questions on a three point Likert scale (agree, not sure, disagree). Scores of 45 and below were considered as poor perception while above 45 was considered as good perception. The data were coded, checked, and processed with version 20 of the Statistical Package for the Social Sciences. Descriptive statistics such as means, standard deviations (SD), frequencies, and proportions were used to summarize variables. Chi-square tests were used to identify associations between categorical variables at 5% level of significance. The University of Ibadan/University College Hospital (UI/UCH) Ethics Review Committee approved the study protocol. Verbal informed consent was obtained from the respondents. The privacy and confidentiality of respondents' data were assured.

## Results

**Figure 1:**
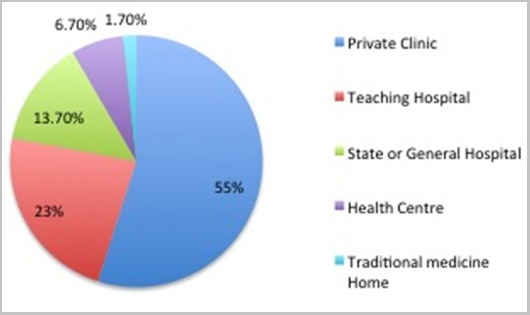
Type of Health facility visited

**Socio-demographic characteristics:** The mean age of the respondents was 39.96 ± 9.1 years. One hundred and fifty (50.0%) of the respondents were males, 242 (80.7%) were married while 54 (18.0%) were single. Majority, 272 (90.7%) reported having a tertiary education, while only 28 (9.3%) had a secondary education. The senior grade level civil servants were 43 (14.3%), while a preponderance of participants were middle grade (7-12) level officers (76.7 %). Majority of the participants (85.0%) were enrolled in the National Health Insurance Scheme (NHIS) and had between 3-5 as household size (49.7%) (Table 1). Overall, 210 (70.0 %) of respondents had ever engaged in self-referral. The most frequently visited types of health facility were the private clinics 165 (55.0%), followed by tertiary health facilities 69 (23.0%) and secondary health facilities 41 (13.7%); primary health centre was visited by 20 (6.7%) participants (Figure 1).

**Table 1:** Socio-demographic Characteristics of participants (n=300)

	N	Percentage (%)
**Gender**		
Male	150	50
Female	150	50
**Marital status**		
Single	54	18.0
Married	242	80.7
Separated	2	0.7
Divorced	2	0.7
**Age Group (in years)**		
18-29	29	9.7
30-39	136	45.3
40-49	73	24.3
50-59	62	20.7
**Level of Education**		
Primary	0	0.0
Secondary	28	9.3
Tertiary	272	90.7
**Income Grade level**		
1-6	27	9.0
7-12	230	76.7
13-17	43	14.3
**NHIS Enrolment status**		
Enrolled	255	85.0
Not Enrolled	45	15.0
**Household Size**		
1-2	93	31.0
3-5	149	49.7
6-8	55	18.3
>8	3	1.0

**Figure 2:**
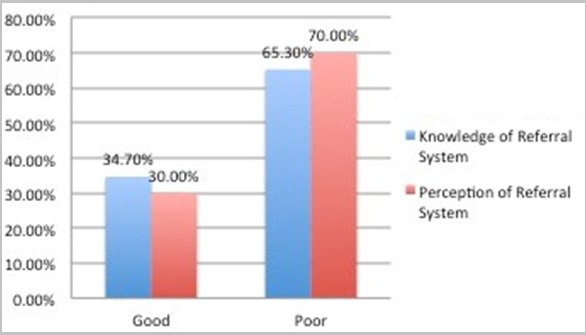
Knowledge and Perception of the Referral System

**Figure 3:**
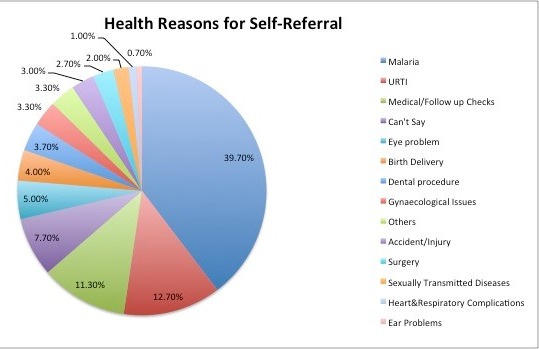
Health reasons for self-referal

**Knowledge and perception of the referral system:** Only 34.7% of the participants demonstrated good knowledge of what a referral system is and its operationalization (Figure 2). Likewise, the proportion of respondents with good perception of a referral system was 30.0% (Figure 2). The most common reasons provided for engaging in self-referrals to higher levels of health care include desire for quality of services (35.7%) and availability of competent staff (35.2%) (Table 2). Other reasons were reputation of hospital (33.8%), location of the hospital (33.3%), past experiences (31.9%), attitude and friendliness of hospital staff members (30.9%) and proximity to place of residence (29.1 %) (Table 2). Malaria was the commonest health problem (39.7%) for which respondents engaged in self-referrals to higher level of health care. Other common health problems reported as reasons for self-referral included upper-respiratory tract infection (URTI) (12.7%), medical and follow-up checks (11.3%), eye problems (5.0%), birth deliveries (4.0%) and dental procedure (3.7%). Less common health reasons for self-referral include gynecological problems (3.3%), accident/injury (3.0%), surgery (2.7%), sexually transmitted diseases (2.0%) and heart/respiratory complications (1.0%) and ear problems (0.7%) (Figure 3). More female respondents (76.0%) compared to male respondents (64.0%) significantly engaged in self-referral (p=0.02, X2 = 5.14). Less respondents with a tertiary education would self-refer compared to those with a secondary education; however, this is not a significant finding (p=0.54, X2 = 0.37). Likewise, respondents who were ever married (p=0.19, X2 = 1.69), currently enrolled in the National Health Insurance Scheme (NHIS) (p=0.60, X2 = 0.28), on income grade levels 1-6 (p=0.44, X2 = 1.66) and got health information with regards to referrals from colleagues (p=0.12, X2 = 5.90) would not engage in self-referrals compared to their counterparts; though these were not significant associations (Table 3). Respondents with good knowledge would not self-refer compared to the respondents with poor knowledge. (p=0.02, X2 = 5.43) (Table 3).

**Table 2:** Reasons for self-referral to higher levels of health care (N = 210)

Reasons for self-referral	N (%)
Quality of Services	75 (35.7)
Qualification/Competence of Staff	74 (35.2)
Reputation of Hospital	71 (33.8)
Location of the hospital	70 (33.3)
Availability of drugs	68 (32.4)
The hospital is under my HMO	67 (31.9)
Past experience with the hospital	67 (31.9)
Affordability of Services	65 (30.9)
Attitude/Friendliness of Staff	65 (30.9)
Proximity to where I live	61 (29.1)
Time spent on queue	49 (23.3)

**Table 3:** Factors associated with self-referral amongst participants (N=300)

Socio-demographic Characteristics	No Self Referral (N=90)	Self-Referral (N=210)	X^2^	p value
**Gender**				
Male	54 (36.0)	96 (64.0)	5.14	0.02^+^
Female	36 (24.0)	114 (76.0)		
**Educational level**				
Secondary	7 (25.0)	21(75.0)	0.37	0.54
Tertiary	83 (30.5)	189 (69.5)		
**Marital status**				
Never Married	20 (58.8)	34 (41.2)	1.69	0.19
Ever Married	68 (28.1)	174 (71.9)		
**NHIS Enrolment status**				
Enrolled	75 (29.4)	180 (70.6)	0.28	0.60
Not Enrolled	15 (33.3)	30 (66.7)		
**Income Grade Level**				
1-6	11 (40.7)	16 (59.3)	1.66	0.44
7-12	67 (29.1)	163 (70.9)		
13-17	12 (27.9)	31 (72.1)		
**Knowledge level**				
Good	40 (38.5)	64 (61.5)	5.43	0.02^+^
Poor	50 (25.5)	146 (74.5)		
**Source of health Information**				
Friends/Family	31 (33.3)	62 (66.7)	5.90	0.12
Colleagues	17 (41.5)	24 (58.5)		
Health workers at the hospital	19 (21.8)	68 (78.2)		
Media	22 (28.6)	55 (71.4)		

**Declarations. Ethics approval and Consent to participate:** Ethical approval for the conduct of the study was obtained from the UI-UCH Ethics committee. Data collection process was performed according to standard ethical guidelines. **Consent for publication:** Not Applicable

## Discussion

Majority of respondents patronised the formal health sector such as private and public health facilities when seeking treatment or care for their health conditions. Authors had reported lower values of 30-40% in similar populations compared to 98.3% obtained in this study [18, 19]. The differences in rural and urban residence of respondents in these studies might have been partly responsible. This proportion is also consistent with findings from a similar study in Enugu [18] (also conducted amongst civil servants) which demonstrated a significance of study participants on the manner, pattern and prevalence that accessed care from formal health care facilities. Utilisation of informal health sector such as traditional medicine homes in this study was expectedly low at 1.7% compared with 10.6% reported by a study in north-western Nigeria [19] where patronage of traditional homes before presentation in a formal health facility was a common practice and accounted for late presentation. Poor knowledge of self-referral was demonstrated by participants in this study is consistent with a similar previous work in Ilorin Nigeria, where 64.8% and 32.0% reported poor knowledge and understanding respectively on the use of primary health care as first point of call for health care service [20]. This lack of understanding is also amplified by findings from a study in Zimbabwe, where a study population did not know the functional differences between a hospital, a clinic and a basic health centre; instead they could only identify the physical differences [17]. From the foregoing, some authors had stressed that the importance of having adequate knowledge on contents and principles guiding management of specific health services and their roles on the referral system cannot be over-emphasised and therefore, should be taken as a complementary when issues of referral are being considered [21, 22]. The desire to obtain care from competent staff as a reason for self-referral in this study is corroborated by findings from literature [23]. Nevertheless, the trend and desire had flouted well-established principles guiding access to care in the study setting [24].

In a study conducted at the University College Hospital, Ibadan Nigeria among medical consultants, 84.1% had good knowledge of the referral system [25]. This high level of knowledge is logical since the respondents were medical consultants whose referral of patients was a crucial part of their daily job description and responsibility. The implication of the above findings is that poor knowledge of referral system will invariably result in misuse or abuse of the health system where health problems that can easily be managed at a lower level of health care are presented at a higher level. A two-way referral system is advocated from the lowest level of health care to the highest, except in emergency situations when patients can be referred to any of the facilities for immediate treatment [26]. This is hardly the case in many developing countries. The practice of by-passing primary health care clinics seems to be driven by a number of factors ranging from the patients' perception of superior quality of care to resource availability in the hospitals; or in other instances, it is simply a case of proximity of a secondary/tertiary hospital to household residences [8]. These assertions were corroborated by our findings as perceived quality of services, qualification of medical staff, reputation and the location of the hospital were given as the main reasons for engaging in self-referral or deciding on the health facility to seek for treatment. Evidence supporting suboptimal quality of services at primary health care facilities abound in the literature [27, 28]. In a study conducted in Tanzania, 44.0% of the women seeking care had by-passed their nearest health facility while 59.8% who lived in a village with a functioning health facility had delivered at home [29]. In the study, the women reported that quality of care (e.g. best provider, availability of drugs) and a greater trust in health workers at the health facility were the main reasons for selecting the facility. Findings of a household survey in Lushoto district in Tanzania showed that patients by-passed their lower level of health care to seek hospital treatment because of poor quality of services and poor availability of drugs [30]. In a similar study in Kenya, respondents considered location of the hospital, good reputation of the hospital, quality of care offered at the hospital, friendly staff and aesthetics of the premises as institutional factors for self-referral [31].

Regulated conditions guiding health insured participants might be a good reason why self-referral was not as high as documented in other studies considering that all the participants in this study were civil servants and a considerable number of them were insured by the current National Health Insurance Scheme (NHIS). In this scheme, enrollee referral can only be approved by an accredited and registered health care provider thus discouraging the practice of self-referrals. This process could be responsible for limiting the rate of self-referral among this group of respondents. Liu et al. (2008) reported that among by-passers of primary care, 5.0% cited health insurance as the reason for seeking care by themselves outside their local community [32]. Some reasons provided for self-referrals included little confidence in the care they would receive at the lower hospitals and lack of well-designed referral system with defined procedures, management support. A potential bias in this study was recall bias which is a usual occurrence with self-reported prevalence surveys. Recall bias was reduced by limiting enquiries on health-seeking behaviour to the last three months. This cut-off has been used widely by researchers in several countries [33, 34]. Another limitation is that the cross-sectional design of this study does not allow inferences to be drawn from its results. Furthermore, the fact that our study only sampled federal civil servants at the federal secretariat greatly limits its generalizability to other populations. Nevertheless, the study provided additional insight into the dynamics and preferences of federal civil servants with regards to access and health seeking behaviours that may be used to project for other federal workers in the country.

## Conclusion

A strengthened health care system translates to access to healthcare services for a country's citizens. Appropriate referral between health facilities is one of the indicators of a strengthened health system. To achieve effective functioning of referral systems, health personnel must be competent and available and the citizens must know the importance of the referral system to avoid potential abuse. This study has shown that civil servants practised self-referral although majority of them were under the NHIS which should be a factor to limit this practice; however, this is not the case. This shows that the National Health Policy and NHIS guidelines on referral are not been effectively implemented. Most of the civil servants showed positive perception of quality of care received despite some domains of dissatisfaction. Domains on staff attitude, time medical staff spend with patients and quality of services at the lower level of care should be improved and the success sustained to avoid dissatisfaction from patients which in turn could push them to access care at the higher health facilities. Improving the quality of care at PHC facilities could also reduce delays in seeking appropriate care. Making simple investigations like rapid malaria diagnostic tests available and securing essential drugs at PHC facilities would improve case diagnosis and management which in turn may increase trust in the care provided at these facilities. It is desirable to ensure that secondary and tertiary health institutions concentrate on their roles as referral centres rather than forced to perform functions of health centres. To realize this, providing the necessary manpower, drugs and facilities would increase the confidence levels of patients in lower level health care facilities. In view of the above, it is suggested that public awareness and education on referral system and services available at different levels of health care should be intensified in order to improve the efficiency of the health systems. Secondly, improving and upgrading the quality of services provided at the lower levels of health care should be a priority for increasing the confidence of the users and reducing its by-pass.

### What is known about this topic

- A good proportion of patients continue to by-pass primary level health care everyday;
- More female patients were engaged in self-referrals however not certain to what extent.

### What this study adds

- The study provides information on common ailments that encouraged self-referrals among federal civil servants in southwestern Nigeria;
- The study establishes a significant association between gender and self-referrals among the participants (being female was associated with increased levels of self-referrals);
- The study also showed that knowledge has a significant role to play in curbing self-referrals amongst populace (In this study, good knowledge of the referral system was associated with lesser amounts of self-referrals).
